# Lipid tethering of breast tumor cells reduces cell aggregation during mammosphere formation

**DOI:** 10.1038/s41598-021-81919-9

**Published:** 2021-02-05

**Authors:** Lekhana Bhandary, Patrick C. Bailey, Katarina T. Chang, Karen F. Underwood, Cornell J. Lee, Rebecca A. Whipple, Christopher M. Jewell, Eleanor Ory, Keyata N. Thompson, Julia A. Ju, Trevor M. Mathias, Stephen J. P. Pratt, Michele I. Vitolo, Stuart S. Martin

**Affiliations:** 1 Marlene and Stewart Greenebaum Comprehensive Cancer Center, University of Maryland School of Medicine (UMGCCC), 22 S. Greene St., Baltimore, MD 21201 USA; 2 Graduate Program in Biochemistry, University of Maryland School of Medicine, 800 W. Baltimore St., Baltimore, MD 21201 USA; 3 Graduate Program in Life Sciences, University of Maryland School of Medicine, 800 W. Baltimore St., Baltimore, MD 21201 USA; 4 UMGCCC Flow Cytometry Shared Service, 655 West Baltimore Street, BRB 7-022, Baltimore, MD 21201 USA; 5 Fischell Department of Bioengineering, 3102 A. James Clark Hall, College Park, MD 20742 USA; 6 Department of Physiology, University of Maryland School of Medicine, 655 W. Baltimore St., Baltimore, MD 21201 USA; 7 Bressler Research Building Room 10-29, 655 West Baltimore Street, Baltimore, MD 21201 USA

**Keywords:** Biotechnology, Cancer, Cell biology, Oncology, Engineering

## Abstract

Mammosphere assays are widely used in vitro to identify prospective cancer-initiating stem cells that can propagate clonally to form spheres in free-floating conditions. However, the traditional mammosphere assay inevitably introduces cell aggregation that interferes with the measurement of true mammosphere forming efficiency. We developed a method to reduce tumor cell aggregation and increase the probability that the observed mammospheres formed are clonal in origin. Tethering individual tumor cells to lipid anchors prevents cell drift while maintaining free-floating characteristics. This enables real-time monitoring of single tumor cells as they divide to form mammospheres. Monitoring tethered breast cancer cells provided detailed size information that correlates directly to previously published single cell tracking data. We observed that 71% of the Day 7 spheres in lipid-coated wells were between 50 and 150 μm compared to only 37% in traditional low attachment plates. When an equal mixture of MCF7-GFP and MCF7-mCherry cells were seeded, 65% of the mammospheres in lipid-coated wells demonstrated single color expression whereas only 32% were single-colored in low attachment wells. These results indicate that using lipid tethering for mammosphere growth assays can reduce the confounding factor of cell aggregation and increase the formation of clonal mammospheres.

## Introduction

The heterogeneity of tumor cell populations is an issue that has become highlighted in recent years. Although the underlying causes of heterogeneity are yet to be completely elucidated, it is clear that some sub-populations in many cancer types possess stem-like properties^1^. Although the theory of cancer stem cells is controversial, it is widely accepted by many that putative cancer stem cells (CSCs) are heavily involved in tumor growth, invasion and metastasis^2^. Investigational work involving these stem-like cells has shown that cancer can arise due to malignant transformation of progenitor stem cells or as a result of dedifferentiation of a cancer cell into a cancer stem cell^3,4^. CSCs, also known as cancer-initiating cells, can self-renew and differentiate to give rise to an entire tumor mass^5^. CSCs can be identified both phenotypically and functionally^4,6^. Labeling cell surface markers such as CD44 (breast)^6^, CD133 (bone)^7^, CD34 (blood)^8^, Lgr5 (colon)^9^ etc., is a phenotypic method for CSC enrichment. Monitoring anchorage-independent growth in vitro or tumor formation in immune-compromised mice in vivo are assays typically used for functional CSC identification^4^*.* Furthermore, a number of studies to have shown that CSCs are resistant to rate-limiting steps of metastasis such as anoikis and can activate gene signatures associated with cell-survival, migration and invasion^10–14^. Finally, cancer cells that induce the epithelial-to-mesenchymal transition (EMT) program involved in early development and migration, have been shown to up-regulate expression of stem-like characteristics such as self-renewal and differentiation^15–18^.

With research in the past decade rapidly highlighting the role of CSCs in tumor initiation and progression, CSCs are now considered prime therapeutic targets for cancer inhibition and/or ablation^19^. Sphere forming assays that monitor the anchorage independent growth characteristics of stem-like cells are a widely employed in vitro model to assess the quantity of CSCs in a bulk population of cancer cells. The propensity of Breast cancer cells to form spheroids (mammospheres) has been linked to stem-like properties and has been shown to be indicative of tumorigenicity in mice^20^. Originally developed as a neurosphere assay to isolate putative stem cells from adult brain cells, the sphere-forming assay has now become a popular choice for identification of stem-like cells from tumor cell-lines and tissue^21^. Mammosphere assays are an in vitro approach used to provide a quantitative estimate of the number of cells that have cancer-initiating capacity in a population of heterogeneous tumor cells. Typically, a tumor cell-line or tissue is processed to obtain single-cell suspensions and 10^3^ to 10^5^ cells per mL are seeded in ultra-low attachment plates, incubated for growth for 5–14 days and spheres that are greater than 40–60 μm in size are counted^20^. Each mammosphere thus formed is believed to be clonal, originating from a single tumor cell. The sphere-forming efficiency (SFE) of a tumor cell line is measured as the number of mammospheres formed relative to the number of single cells originally seeded. However, the SFE calculated in this manner does not effectively account for aggregation of the free-floating cells into clusters. These aggregates can confound the true sphere-forming efficiency of tumor cells. Stem cells have been shown to form aggregates in suspension-culture, which promotes their survival and pluripotency^22,23^. Moreover, aggregate-mediated enhancement of stem cell properties as a result of reprogramming into stem-like cells has been reported in non-adherent culture of several non-stem cell types including fibroblasts, ocular epithelial cells, and human embryonic kidney epithelial cells^23–25^. Cell aggregation in non-adherent culture conditions is routinely used to generate stem cells^26–31^. While aggregation-mediated stem cell enhancement is favorable when trying to culture stem cells in vitro, it interferes with the isolation of tumor cells with cancer stem cell potential when the goal is identification of self-renewal and clonality.

To address these shortcomings, we developed a technique to minimize tumor cell aggregation after seeding but still allowing for higher-throughput. Our technique reduces free cell movement yet retains a non-adherent microenvironment. This enables those tumor cells with stem-like potential to form mammospheres while reducing cell aggregate formation. To create a low attachment surface, tissue culture-treated plates were coated with alternating layers of polyelectrolytes. Polyelectrolyte multilayers (PEM) are assembled through electrostatic or hydrogen-bonding interactions between layers to form a surface that inhibits cell attachment. The PEM surface is then coated with a charged lipid, creating a hydrophobic tether which immobilizes cells in space. Lipid Tethered cells retain the characteristics of cells in suspension due to the non-adherent PEM layer beneath. A number of published reports have demonstrated the utility of coating surfaces with PEM and using lipids or other binding moieties to create cell-binding tethers in a low-attachment environment^32–36^. A recently published report from our laboratory demonstrated that microfluidic substrates coated with PEM plus DOTAP lipid moieties successfully tethered breast tumor cells and maintained their free-floating behavior even after a number of fluid washes^37^. In addition, the lateral drift of tethered cells was dramatically reduced^38^.

Recent literature has highlighted the problem of cellular aggregation in the mammosphere assay^39,40^ and we propose here a more high-throughput solution to the problem. Single cell sorting using FACS has been used successfully to generate tumorspheres^41^. This technique however necessitates that every well be visually inspected to ensure it contains a cell. If every well is not inspected, results will be contaminated by false negatives. Single cell sorting also requires hundreds of plates to achieve the throughput our technology can achieve in a single plate. The Limiting Dilution Assay suffers the same drawbacks. It is low throughput, requires visual inspection of every well and wells with higher dilutions will contain aggregations. Aggregations contaminate sphere forming efficiency calculations. We show that using a PEM + lipid coated surface reduces cell aggregation when compared to ultra-low attachment plates and that singly tethered cells form mammospheres similar to those observed under ultra-low attachment conditions. Furthermore, size quantification of mammospheres indicates that PEM + lipid coated surfaces yield smaller mammospheres consistent with the size of spheres formed from single cells sorted with flow cytometry.

## Results

### PEM + lipid coated plates can be used for mammosphere assays in lieu of ultra-low attachment plates

**Figure 1:**
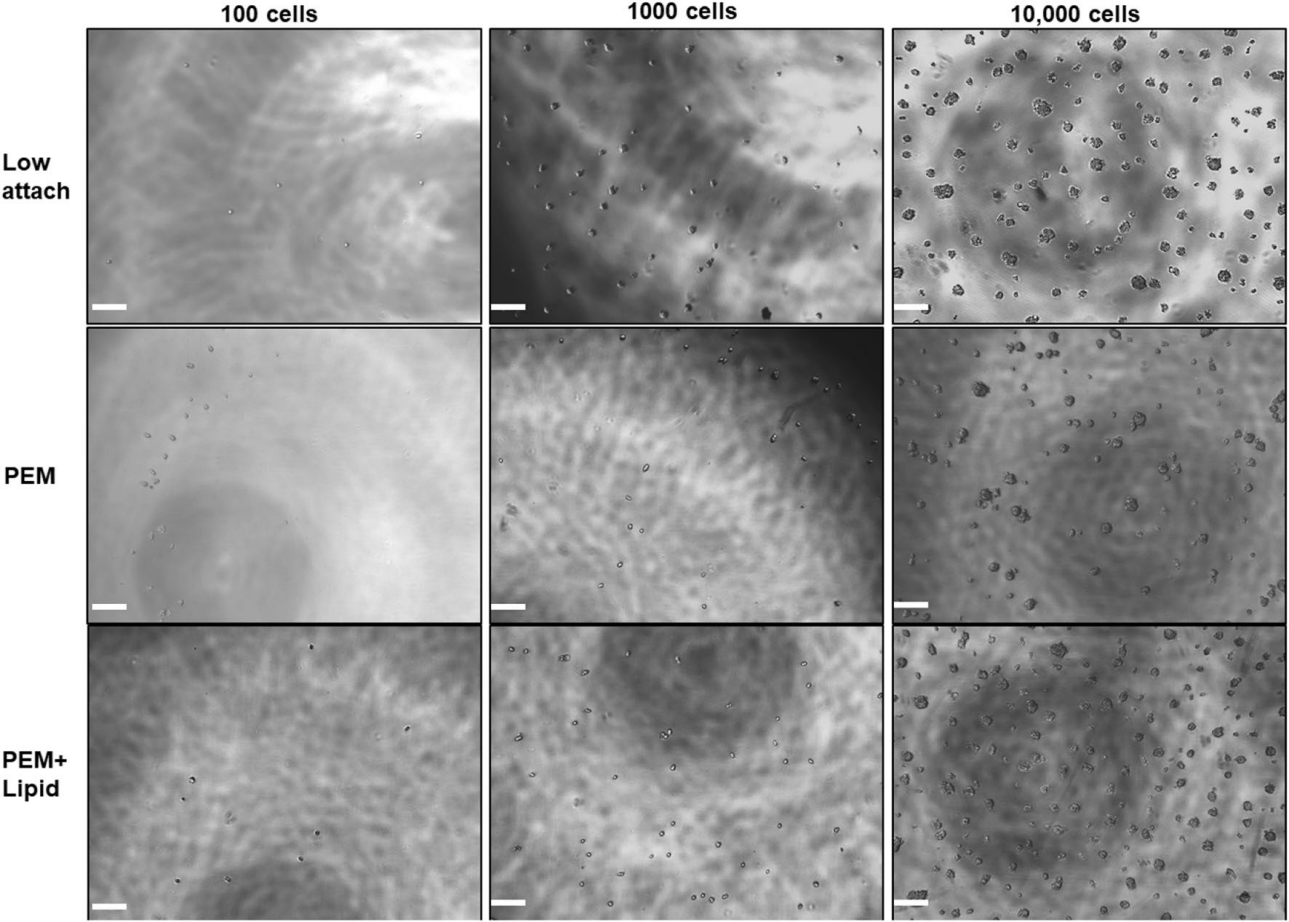
PEM + lipid coated plates can be used for mammosphere formation: FACS was used to seed single cell suspensions of MCF7 cells into 96 well low attach plates with the indicated surface treatments. Left column—100 cells/mL. Middle column—1000 cells/mL. Right column—10,000 cells/mL. Phase contrast images of resultant mammospheres for Day 1 (24 h) post seeding are shown. (×4 magnification, bar 200 µm).

There is no standardized seeding density employed by the field for mammosphere formation. Densities ranging from 10^3^ to 10^5^ tumor cells per mL have been reported^18^. To analyze the effect of seeding density on aggregate formation on a lipid anchored surface, we seeded either 100 cells/well (333 cells/mL, 312.5 cells/cm^2^) or 1000 cells/well (3333 cells/mL, 3125 cells/cm^2^) into ultra-low attachment 96-well plates as well as 96-well plates coated with either PEM or with PEM + lipid. The PEM coating creates a surface that prevents cell attachment, while the lipid coat provides a tether which anchors the cell membrane but does not allow cells to spread on the surface^37^. We also seeded 10,000 cells/well (33,333 cells/mL, 31,250 cells/cm^2^) to visualize the effect of the higher seeding densities often employed in mammosphere assays. To prevent doublets or clustered cells from being added at the time of initial seeding, flow cytometry was used to sort single cell suspensions of MCF-7 cells. Our lab has recently shown that pre-spheres (PreSp) grown from single cells should not be larger than 4 cells at 24 h^39^. Visual inspection of phase contrast images taken on Day 1 post-seeding show that the MCF7 PreSp present in PEM + lipid coated wells seeded with either 100 or 1000 cells generally conform to this criteria (Fig. 1, left and center columns). However, despite the use of flow sorted single cell suspensions, wells seeded into plain low attach plates at densities of 1000 and 10,000 cells showed the presence of clusters over 4 cells in all plates, in all wells on Day 1. Larger clusters are especially evident in wells seeded with 10,000 cells in both low-attach and PEM + lipid wells. This indicates that clumping is directly proportional to seeding density due to space constraints (Fig. 1, right column). Therefore, most of the mammospheres that ensue from seeding such high densities are not single cell clones, but rather the results of cellular aggregation. Furthermore, within each plate type, spheres formed on Day 7 were much smaller in wells seeded with either 100 cells/well or 1000 cells/well compared to wells seeded with 10,000 cells (Supplemental Figure S1A). Taken together with Day 1 cluster size, this size data indicates that the spheres observed in wells seeded with 10,000 cells are cellular aggregates as opposed to clonal growths. Additionally, spheres greater than 60 μm in size are formed in all conditions on days 7 and 14, indicating that PEM or PEM + lipid coating does not hamper mammosphere formation (Supplemental Figure S1A and B).

### PEM + lipid coated wells generate mammospheres with greater efficiency and smaller size compared to low attachment wells and methylcellulose

**Figure 2:**
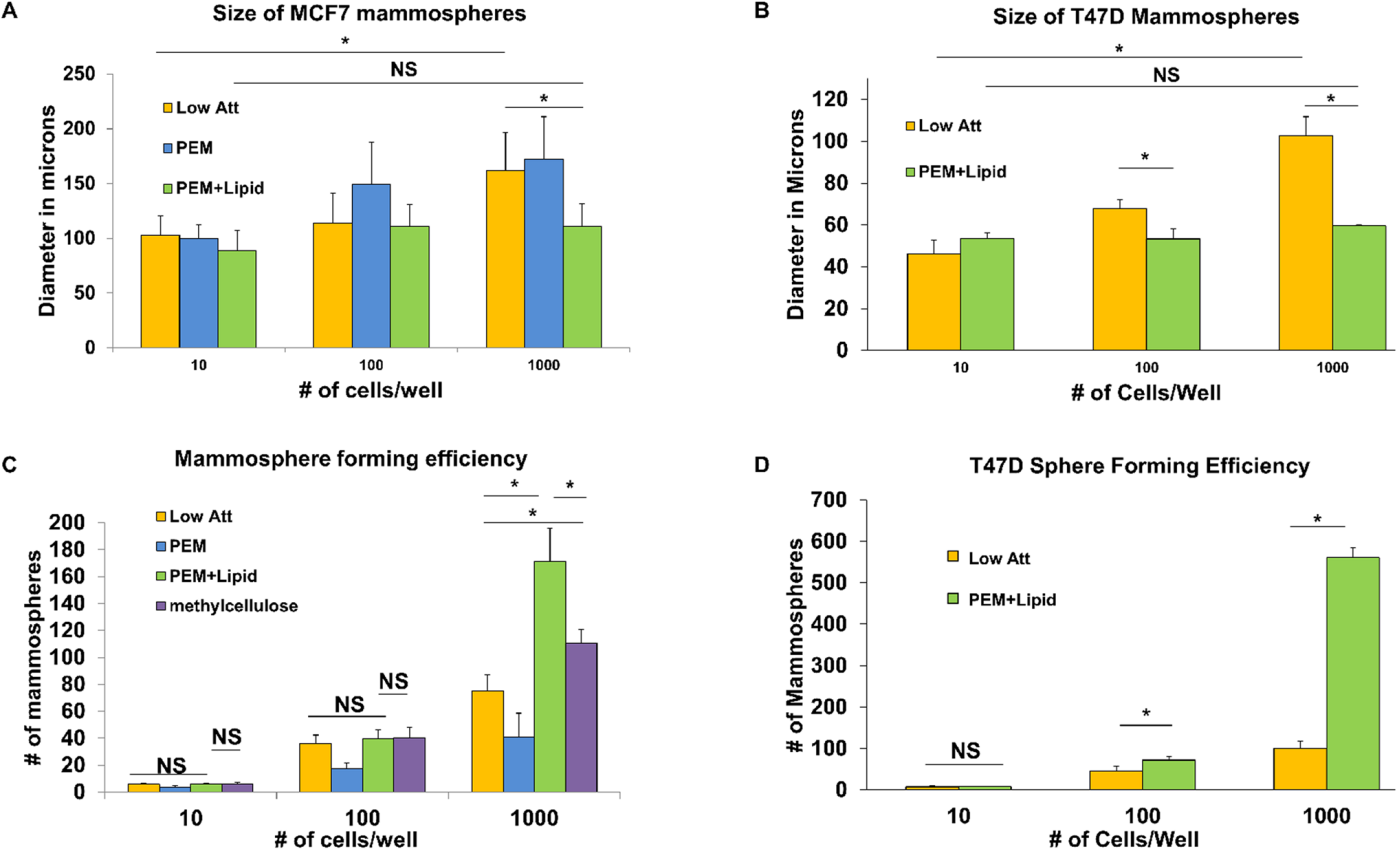
PEM + lipid coated wells generate mammospheres with greater efficiency and smaller size compared to low attachment wells: for (**A**,**C**) FACS was used to seed single cell suspensions of MCF7 cells at densities of either 10, 100 or 1000 cells into low attach and PEM + lipid coated well plates. For (**B**,**D**) T47D cells were employed. (**A**,**B**) At low seeding densities of 10 cells/well or 100 cells/well, the average size of mammospheres formed is comparable in low attach and PEM + lipid wells. At a seeding density of 1000 cells/well, there are significantly different averages of spheres in the low attach and PEM + lipid wells, respectively. (**C**) The total number of mammospheres formed in low attach and PEM + lipid coated wells have no significant difference when 10 MCF7 cells/well or 100 MCF7 cells/well are seeded. However, when 1000 cells/well were seeded, there were significantly different averages between the low attach and PEM + lipid coated wells. (**D**) The total number of mammospheres formed in PEM + lipid coated wells have no significant difference when 10 T47D cells/well are seeded. However, when 100 or 1000 cells/well were seeded, there were significantly different averages between the low attach and PEM + lipid coated wells. (×40 magnification, bar 50 μm). Statistics formulated using ANOVA with post-hoc Tukey HSD. *p value < .01.

In order to evaluate the effects of seeding density dependent aggregation on SFE, we analyzed the size and quantity of mammospheres in low attachment plates versus PEM + lipid coated plates by seeding either 10, 100, or 1000 cells/well in 96-well plates. Methylcellulose has been reported to reduce cellular drift and resultant aggregation^16,42^. We sought therefore to compare our technology to established procedure to determine its comparative efficacy. The percentage of methylcellulose used is publication dependent, so we utilized the highest percentage we found reported^43^ to provide the most reduction of aggregation. T47D and MCF7 cells were seeded at either 10, 100 or 1000 cells/well. We found that the average size and number of spheres formed at Day 7 is not significantly different between low-attach and PEM + lipid wells at the two lowest densities of MCF7 cells (Fig. 2A,C).

This indicates that mammosphere size and efficiency is not confounded by aggregation when cells are seeded sparsely. However, seeding 1000 cells/well revealed a significant difference in the SFE of MCF7 cells. MCF7s seeded at 1000 cells/well yielded a mean of 75.2 spheres in the low attachment wells (SFE 7.5%), whereas an average of 171.2 spheres (SFE 17.1%) formed in the lipid-coated wells (Fig. 2C). Increasing the number of cells seeded in a well decreases the surface area/cell for free movement, resulting in more cellular aggregates in the low attachment plates and a correspondingly lower SFE. In wells coated with PEM + lipid, individual cells were immobilized and therefore physically prevented from aggregation through lipid tethering. Although a significant increase of efficiency compared to low-attach plates was observed with methylcellulose, it did not reduce aggregation to the extent of PEM + lipid. Methylcellulose wells had an average of 110.3 spheres (SFE 10.3%, Fig. 2C). To visualize the ability of cells to move even in methylcellulose we imaged an entire well at day 0 and at 24 h (Supplemental Figure S2A), illustrating clear cellular drift. At 24 h cells are already beginning to build up at the edges of the wells (Supplemental Figure S2B). Examining average sphere size vs. seeding density (Fig. 2A) demonstrates that the average sphere size increases significantly with greater seeding density in the low attachment wells, but spheres in lipid-coated wells show no significant change in size with any density. In wells seeded with 1000 cells, spheres in PEM + lipid wells were significantly smaller than spheres in low attach wells (Fig. 2A). T47D cells however, require a very low seeding density to negate the effects of aggregation. A significant difference in size and SFE is already apparent at a density of 100 cells/well (Fig. 2B,D). This result conforms to our earlier published work that indicates T47D cells have a higher propensity to drift to the edge of wells and form aggregations than MCF7 cells^39^. Altogether, by preventing aggregation, the lipid-coated wells had an increased number of mammospheres that were on average smaller in size when compared to mammospheres that formed in the low attachment wells. Higher magnification imaging showed that tethered cells form mammospheres that are morphologically similar to those formed in the traditional ultra-low attachment plates (Supplemental Figure S1C).

### Lipid tethering reduces the effects of aggregation

**Figure 3:**
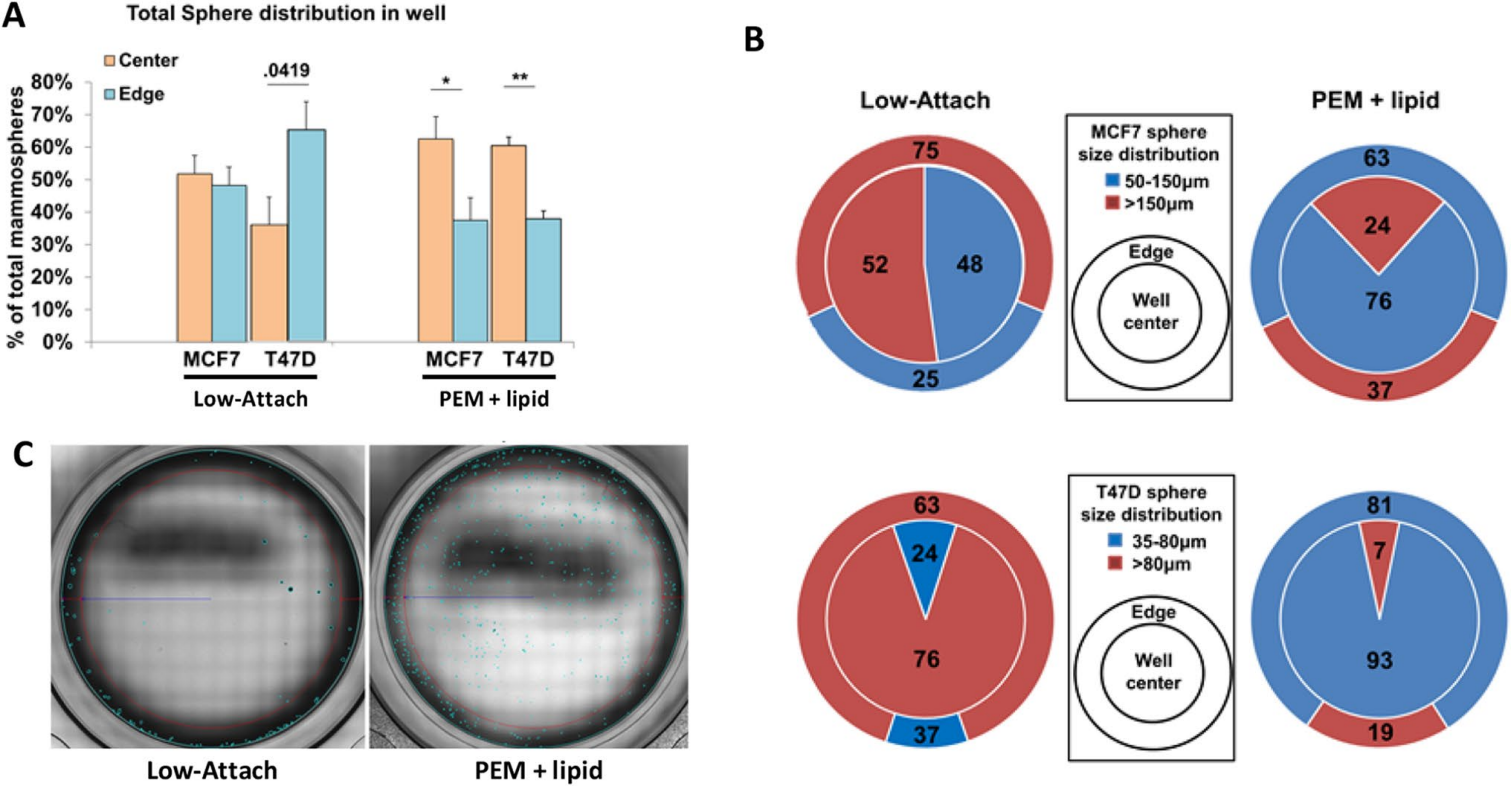
Lipid tethering reduces the effects of aggregation: single cell suspensions of MCF7 and T47D cells were used to seed 1000 cells into low attach and PEM + lipid coated well plates and were analyzed on Day 7 post-seeding. (**A**) Low attachment wells have no significant difference in distribution of mammospheres in the well center and at the well edge for either MCF7 or T47D cells, whereas lipid coated wells have a significant difference of > 20% between spheres in the center and edges of the well in both cell lines. (**B**) Panel (**B**) is a visual representation of sphere size distribution in the wells. Edge size distribution at the edge is displayed in the outer ring. Well center distribution is displayed in the inner circle. Values are in percentages. (**B**, upper) Low attach plates have a nearly equal size distribution in the center of the well, with the edge having a 75% (± SD6) population of spheres over 150 μm. PEM + lipid wells have only 36% (± SD4) spheres over 150 μm at the well edge and 24% (± SD3) in the center. (**B**, lower) Representative images of T47Ds greater propensity to form aggregations, low attach plates have higher concentrations of spheres > 80 μm in both the well center and well edge. PEM + lipid plates however have 93% and 81% clonal spheres in the center and at the edge, respectively. (Statistics formulated using ANOVA with post-hoc Tukey HSD. *p value < .01, **p < .001) C) Representative images of wells seeded with 1000, T47D cells in either low attach or PEM + lipid plates and imaged on day 7. Red circle marks 500 μm from the well edge.

Cellular drift towards the plate edge is a regular occurrence in the mammosphere assay. In order to evaluate if coating the well surface with lipid reduces this movement, FACS was used to seed 1000 MCF7 cells/well into low attachment and PEM + lipid coated 96-well plates. On Day 7 post-seeding, mammospheres that were touching the well edge or within 500 μm distance from the edge of the well were marked as edge positive, and the remaining spheres were marked as being centered. Figure 3A shows 51.7% of MCF7 mammospheres in the well center and 48.2% at the well edge. Although this seems like an even distribution, it is important to note here that the area we have defined as edge positive is 2.5 times smaller than the area defined as centered and makes up only 29% of the total well area. Nearly half the spheres are concentrated in a small area near the edge. Inspection of the lipid coated wells reveals more spheres in the center of the well (62.5%) and less at the edge (37.5%). T47D cells were also seeded at 1000 cells/well and sphere distribution was analyzed in the same manner. In low attach wells however, T47D spheres showed a greater propensity to drift to the edge. 65.5% of T47D spheres were edge positive in low-attach plates. The PEM + lipid plates effectively reversed this trend with 61.2% of spheres located in the center (Fig. 3A). Given that we observed a difference in the size of the spheres that were being formed in the low attachment and lipid-coated plates (Fig. 2A,B) we sought to establish an average size of verifiably clonal spheres. To this end, we used FACS to seed single cells into each well of a 96-well low attachment plate and measured the size of mammospheres that were formed. The average size of the resultant mammospheres on Day 7 post-seeding was 106 µm. We therefore classified mammospheres into two categories: those between 50 and 150 μm (clonal) in size and those greater than 150 μm (aggregates). Our recent single cell tracking data also verifies values over 150 μm for MCF7 spheres are aggregations^36^. Using this categorization to analyze the entire well, we observed that in the low attachment plates 37% of MCF7 spheres were between 50 and 150 μm in size, whereas 63% of the spheres were greater than 150 μm in size. On the other hand, in the lipid-coated wells, 71% of the spheres were between 50 and 150 μm and 29% were greater than 150 μm in size indicating significantly reduced aggregation (Supplemental Figure S3B). For T47D cells we defined clonal spheres between 35 and 80 μm as previously published^39^. The differences in T47D clonal sphere distribution were even more pronounced. In Low attach plates T47D aggregations comprised 81.9% of the total sphere population. This phenomenon was again effectively reversed by our technology. In PEM + lipid plates spheres > 80 μm made up only 18.1% of the total sphere population, a reduction of four fold (Supplemental Figure S3A). Further size analysis with respect to well location revealed an almost equal allocation of spheres between 50 and 150 μm in size (48%) and those greater than 150 μm in size (52%) in the well center (Fig. 3B, upper). Conversely, in the center of the lipid-coated wells, 76% of the spheres were between 50 and 150 μm and only 24% were greater than 150 μm (Fig. 3B, lower). Well edge analysis indicated that in the low attachment wells only 25% of the spheres were between 50 and 150 μm in size, while the majority of the spheres (75%) were greater than 150 μm. Presumably, more aggregates will form at the well edges where the movement of cells becomes physically limited and cells begin to accumulate. However, this trend was not observed at the edge of the lipid-coated wells, where 63% of the spheres were between 50 and 150 μm in size while only 37% were greater than 150 μm (Fig. 3B, upper). The decrease in the proportion of spheres greater than 150 μm at both the center and edge of the lipid-coated wells compared to the low attach plates indicates that cell tethering reduces aggregation and allows for the increased formation of clonal mammospheres. Size/location analysis of T47D cells revealed their greater tendency to drift the edges of the well in greater detail. Spheres > 80 μm made up the bulk of the total sphere population in both the center and edges of the wells. Strikingly, aggregations were nearly ablated in the center of PEM + lipid plates with only 7% of spheres over 80 μm remaining (Fig. 3B, lower). Figure 3C displays stitched images of an entire well to visualize the morphological/spatial differences of spheres between low attach and PEM + lipid coated plates. Primary spheres are easily removed from the PEM + lipid surface by pipetting, and can be used to form secondary spheres (Supplemental Figure S4). In the second generation, T47D cells formed fewer spheres, which is an established characteristic of the T47D cell line^44^, while the MCF7 cells did not show a significant difference between primary and secondary sphere formation.

### Lipid-coated wells have a higher proportion of clonal mammospheres compared to low attachment wells

**Figure 4:**
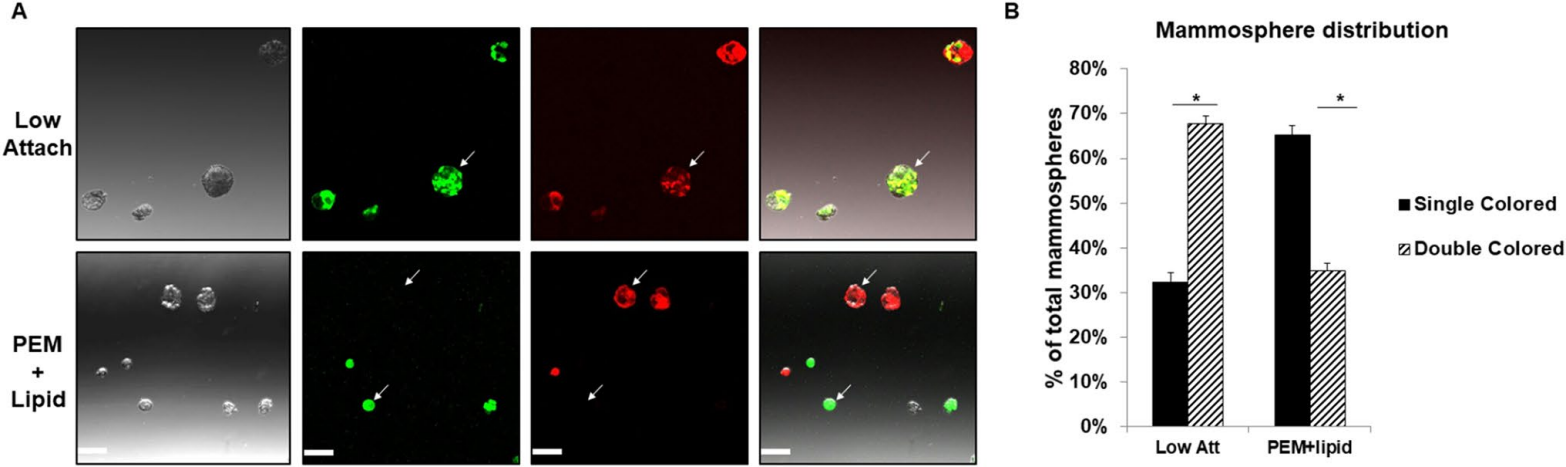
Lipid-coated wells have a higher proportion of clonal mammospheres compared to low attachment wells: (**A**) FACS was used to seed single cell suspensions of MCF7-GFP and MCF7-mCherry cells into 96 well plates. 500 cells of each cell type were sorted together into each well of low attachment and lipid-coated plates. Confocal imaging of mammospheres on Day 7 post-seeding shows that mammospheres in the low attachment plates are positive for both GFP and mCherry expression (top panels, white arrows), whereas the majority of the mammospheres observed in the lipid-coated plates are positive for either GFP expression or mCherry expression (lower panels, white arrows)—×10 magnification, scale bar 200 μm. (**B**) Quantification of mammospheres greater than 60 μm indicates that low attachment wells have significantly more double-colored mammospheres whereas PEM + lipid wells have significantly less. Statistics formulated using ANOVA with post-hoc Tukey HSD. *p value < .01.

To confirm that mammospheres formed from higher seeding densities are the result of aggregation and not clonal growth, we used FACS to seed an equal number of MCF7-GFP and MCF7-mCherry cells together into both 96-well low attachment and lipid-coated plates. Confocal imaging of mammospheres on Day 7 post-seeding showed that the majority of mammospheres in the low attachment plates were positive for both GFP and mCherry expression, indicating that these spheres were clearly formed from MCF7-GFP and MCF7-mCherry aggregates. The majority of mammospheres observed in the lipid-coated plates however, were positive either for only GFP or only mCherry expression (Fig. 4A). Analyzing all mammospheres greater than 60 μm indicated that in the low attachment wells 68% of spheres were positive for both GFP and mCherry expression while 32% contained only a single marker. In the lipid-coated wells, this trend was reversed with 35% of the spheres expressing dual markers and 65% of the spheres having only a single color (Fig. 4B). These data indicate tethering of cells to a lipid coated surface reduces cellular aggregation and allows increased growth of clonal spheres.

## Discussion

Research in the past decade has shown that cancer stem cells play pivotal roles in tumor initiation and progression of many cancer types. The ability to target these cells is important to successful cancer therapy. However, due to multiple inherent properties, CSCs are often resistant to chemotherapeutic drugs^45–47^. Radiation therapy can also be ineffective on CSCs that have highly activated DNA repair pathways^48–50^. Moreover, these cells can also evade immune detection because of down-regulation of MHC-class I and tumor-specific antigens, rendering immunotherapy ineffective^51,52^. This inherent ability of CSCs to evade destruction by currently available cancer therapeutics promotes the survival of minimal residual disease that leads to metastatic recurrence after remission^53–55^. Given the difficulties of targeting CSCs using traditional modes of cancer therapy, research is now being focused on identifying drugs that can selectively target CSCs. However, the first step in this direction is to be able to correctly identify cancer stem cells from a tumor mass. Mammosphere formation is considered an indicator of a stem-like phenotype in tumor cells and mammosphere assays are regularly used to isolate and culture cancer-initiating cells from a heterogeneous mixture of tumor cells. The SFE of breast cancer cells is believed to be an indicator of single tumor cells ability to self-renew. According to this theory, a mammosphere should arise from a single cell. In practice however, many techniques employed to test clonal self-renewal overlook the fact that when cells are seeded at high densities on low attachment plates, they can aggregate and form clusters due to plate handling causing cell movement^56^ or simple overcrowding. Aggregate-mediated enhancement of stem-cell characteristics^23–25^ could influence in vitro cancer stem cell analysis in mammosphere assays, which might not reflect true cancer-initiating properties of tumor cells. Moreover, these aggregates could also be misrepresented as mammospheres and provide incorrect information on the stem-like properties of tumor cells and therefore complicate reproducibility, conclusions and future studies.

To reduce the cell movement and aggregation of suspended cells observed in low attachment plates, we used plates coated with polyelectrolyte multilayers (PEM) to prevent cells from attaching and spreading on the plate surface, and the charged lipid DOTAP to tether individual cells through lipid-cell membrane interactions. A previous publication from our laboratory demonstrated the efficacy of using PEM + lipid coatings to tether breast tumor cells for microtentacle analysis^37^. Here we show that this technology can be extended to mammosphere assays to reduce cellular aggregation in suspension culture and increase numbers of truly clonal mammospheres. This technique is more high-throughput than assays that utilize a single cell per well. To assess the sphere forming capability of 24,000 cells it would take over 240, 96 well plates. PEM + Lipid technology can examine 24,000 cells in one 24 well plate. We also compared this technology with the established use of methylcellulose to determine feasibility and efficacy.

Seeding density can affect cell clustering and aggregate formation, with more clusters being observed at higher cell densities^39,40^. We demonstrated that at very low cell densities of 10 cells/well or 100 cells/well of a 96-well plate, there is sufficient surface area/cell to prevent cell aggregation. Therefore, the size and number of mammospheres formed in low attachment plates, in methylcellulose and the lipid-coated wells is similar. However, at a seeding density of 1000 cells/well, the low attachment and methylcellulose wells show a lower number of mammospheres compared to lipid-coated wells. This result was initially surprising, as we had predicted low attachment wells to have a higher mammosphere forming efficiency due to increased cell aggregation. However, analysis of the sizes of the mammospheres formed indicated that low attachment wells contained spheres with an average diameter of 162 μm whereas those in the lipid-coated wells were on average 111 μm in size. We observed that lipid-coated wells had more mammospheres that were on average smaller in size than those observed in the low attachment wells. This is explained simply by the idea that cells/spheres aggregating together create one larger sphere, increasing average size and reducing SFE. This concept is not unknown in the field, as Shaw and colleagues have published on the concept of using a dilution series to determine the highest usable seeding density sans aggregation^40^. It has also been established that even when methylcellulose is used, the concentration of breast tumor cells must be kept below 1000 cells/mL to prevent aggregation^43^.

Using the size of mammospheres formed from single sorted cells and our previously published data as a guideline for the size of truly clonal mammospheres, we found that MCF7 spheres in the size range of 50–150 μm and T47D spheres in the range of 35–80 μm are most likely to have formed from a single cell, while mammospheres larger than these ranges are likely a result of cell aggregation. Based on this categorization, our technology doubles the amount of clonal MCF7 spheres and increases clonal T47D spheres by fourfold. Further analysis of mammospheres in the low attachment and lipid-coated wells based on their location show that lipid-coated plates increase the proportion of mammospheres in the center of the compared to the edge. This difference is due to the tethering of individual cells in lipid-coated plates, which keeps more cells in the center of the well and prevents them from rolling to the edges. Spatial analysis with regards to sphere size revealed that MCF7 spheres at the edges of low attach wells contained 74.6% aggregates. This number was cut nearly in half in PEM + lipid plates. Strikingly, T47D cells had a strong tendency to move to the edges of plates. Overall, 80% of T47D spheres were found at the edges of low attach wells, and of these 63% were over 80 μm. In PEM + lipid plates only 18% of spheres were found at the edge and these spheres were 81% clonal. T47D spheres in the center of the plate were 93% clonal demonstrating a high efficiency of cell tethering. These differences in size and distribution indicate that tethering reduces cell aggregation even at the edges of the well when compared to low attachment wells. Finally, a clear demonstration that cell aggregation occurs to a greater extent in the traditional low attachment wells was the presence of a higher proportion of mammospheres that expressed both GFP and mCherry, when an equal number of red and green cells were seeded. By comparison, lipid-coated plates showed a higher proportion of single-colored spheres, indicating a greater probability of clonal origin.

These results demonstrate that using this cell tethering technique for mammosphere growth assays can reduce formation of cell aggregates and increase formation of truly clonal mammospheres that arise from a single tumor cell. Furthermore, tethering individual cells also potentially extends the scope of traditional mammosphere assays to experiments such as tracking the early development and fate of cells in real-time as they divide to form mammospheres. Other possible experiments include conducting drug wash-in/wash-out experiments without displacing cells, to determine individual cancer stem cell responses to drug treatments.

## Materials and methods

### Cell culture and reagents

MCF7 and T47D cells were cultured in DMEM (Corning Cellgro #10-017-CV) with 10% fetal bovine serum (Atlanta Biologicals #S11150H) and 1% Penicillin/Streptomycin (Gemini #400-109) and maintained at 37 °C in 5% CO_2_. Stably expressing MCF7-GFP were generated as described previously^57^ and maintained with 0.8 mg/mL Geneticin (Invitrogen #10131027). To make stably expressing MCF7-mCherry clones, MCF7 cells were transfected using ExGen 500 in vitro transfection reagent (Thermo Scientific #R0511) with pmCherry-C1 (Clontech, Mountain View, CA, USA). Stable clones were isolated following Geneticin selection (0.8 mg/mL) and verified by positive fluorescent expression.

### Flow cytometry cell sorting

Cells were trypsinized, counted and 1 × 10^7^ cells were transferred into Falcon round bottomed tubes (Corning, # 352058). The cells were washed in PBS twice with PBS, collected by centrifugation at 1000 rpm for 5 min, and blocked in Flow Incubation Buffer (0.5% BSA in PBS) for 10 min followed by staining with Propidium Iodide (Sigma Cat # P4864) at 0.05 mg/mL for 10 min in the dark according to manufacturer's instructions. Cells were rinsed once with PBS, collected by centrifugation at 1000 rpm for 5 min, resuspended in PBS and passed through Falcon Tube with Cell Strainer Cap (Corning, # 352235) and sorted using BD FACS ARIA II Cell Sorter. For sorting the MCF7-GFP and MCF7-mCherry cell lines, which stably express GFP and mCherry respectively, all steps described above except the incubation and staining steps were performed prior to sorting.

### Mammosphere culture

Cells were trypsinized, triturated repeatedly and passed through a 40um cell strainer to enrich for single cells^39^. Cells were counted and visualized on a hemocytometer^39^. If cell clumps were observed, cells were passed through a 25-gauge needle 10 times^39^. FACS was used to sort cells into Ultra-Low Attachment 96-well plates (Corning #3474) or Tissue-culture treated 96-well plates (Corning 3596) coated with PEM or PEM + lipid. Wells were filled with 300 µL Mammocult media supplemented with heparin, penicillin/streptomycin and hydrocortisone per manufacturers instruction (complete media) The spheres were imaged at Day 7 and Day 14 and spheres greater than 60 µm in diameter were counted as mammospheres and included in analysis. For secondary sphere formation, gentle pipetting ensured dissociation of all spheres from the lipid tethers. Spheres were then trypsinized for 20 min at 37 °C and dissociated into single cells by gentle trituration and passaged through a 40 μm strainer to separate into a single-cell suspension, and then replated onto PEM + lipid surfaces to measure secondary sphere formation.

### Polyelectrolyte multilayer (PEM) and lipid coating

Tissue culture treated 96-well plates (Corning) were primed with 120 µL of 0.047 M pH 3 Polyallylamine hydrochloride (Alfa Aesar #43092) solution for 15 min followed by a wash step with pH 3 water for 1 min. To create a polyelectrolyte bilayer, 100 µL of 0.01 M pH3 Polymethacrylic acid (Polysciences #0058) was added for 5 min, followed by two wash steps, the addition of 100 µL of 0.01 M pH 3 Polyacrylamide (Polysciences #02806) for 5 min, and two more wash steps. A total of four such bilayers were added to create a PEM coated surface. PEM coated plates were allowed to dry overnight. For lipid-coated plates, at the end of PEM coating, 75 µL of 0.01 M DOTAP (Avanti #890890) was added for 5 min, followed by two wash steps. To enable lipid crosslinking, 100 µL of 3.7% formaldehyde (pH3) in PBS was added to the wells for 5 min and followed with two wash steps. All excess fluid was aspirated from the wells and the plates were allowed to air dry for 1 h at room temperature.

## Supplementary Information

Supplementary Information

## Data Availability

Materials and data will be made available upon request as outlined for Scientific Reports-http://www.nature.com/srep/journal-policies/editorial-policies#availability.
