# Supplementary material for: Lipid tethering of breast tumor cells reduces cell aggregation during mammosphere formation
Source: Sci Rep. 2021 Feb 5;11:3214. doi: 10.1038/s41598-021-81919-9 (PMC7865010; doi:10.1038/s41598-021-81919-9)
Supplement: Supplementary file 1 — Supplementary Information [file 41598_2021_81919_MOESM1_ESM.docx]

**Supplementary Information**

**Title:** Lipid tethering of breast tumor cells reduces cell aggregation during mammosphere formation

**Authors:** Lekhana Bhandary^1*^, Patrick C. Bailey^1*, 2^, Katarina T. Chang^1, 3^, Karen F Underwood^4^, Cornell J. Lee^1^, Rebecca A. Whipple^1^, Christopher M. Jewell^5^, Eleanor Ory^1^, Keyata N. Thompson^1,^ Julia A. Ju^1^, Trevor M. Mathias^1^, Stephen J.P. Pratt^1,2^, Michele I. Vitolo^1, 2, 6, 7^ and Stuart S. Martin^1, 2, 6,*^


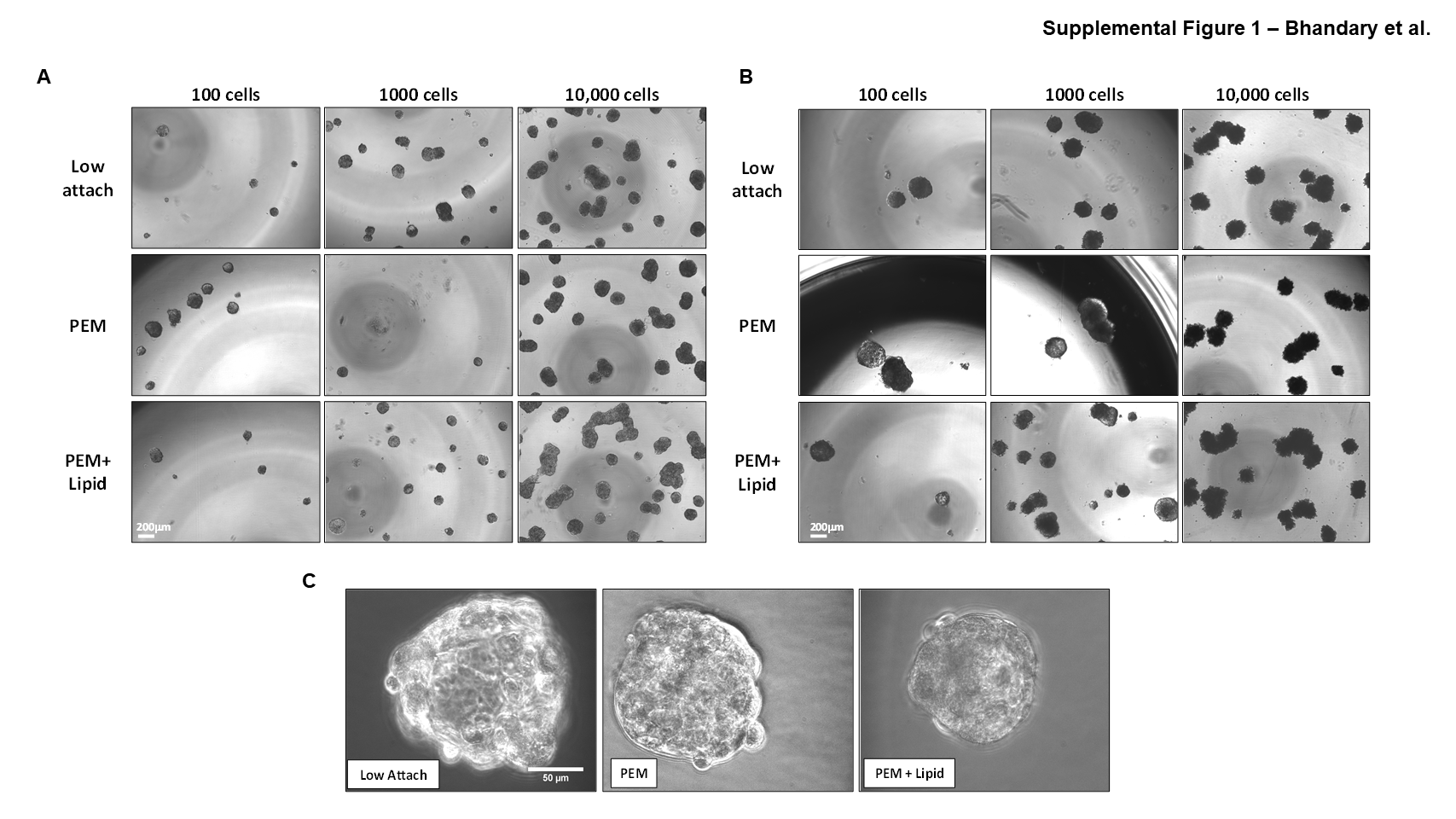


**Supplemental Figure 1:** Long-term sphere formation at different cell densities: FACS was used to seed single cell suspensions of MCF7 cells into 96 well low attach plates with the indicated surface treatments. Three densities of 100, 1000 and 10,000 cells were used. Phase contrast images of resultant mammospheres are shown for Day 7 (a) and Day 14 (b) at 4x magnification (Bar=200 µm).


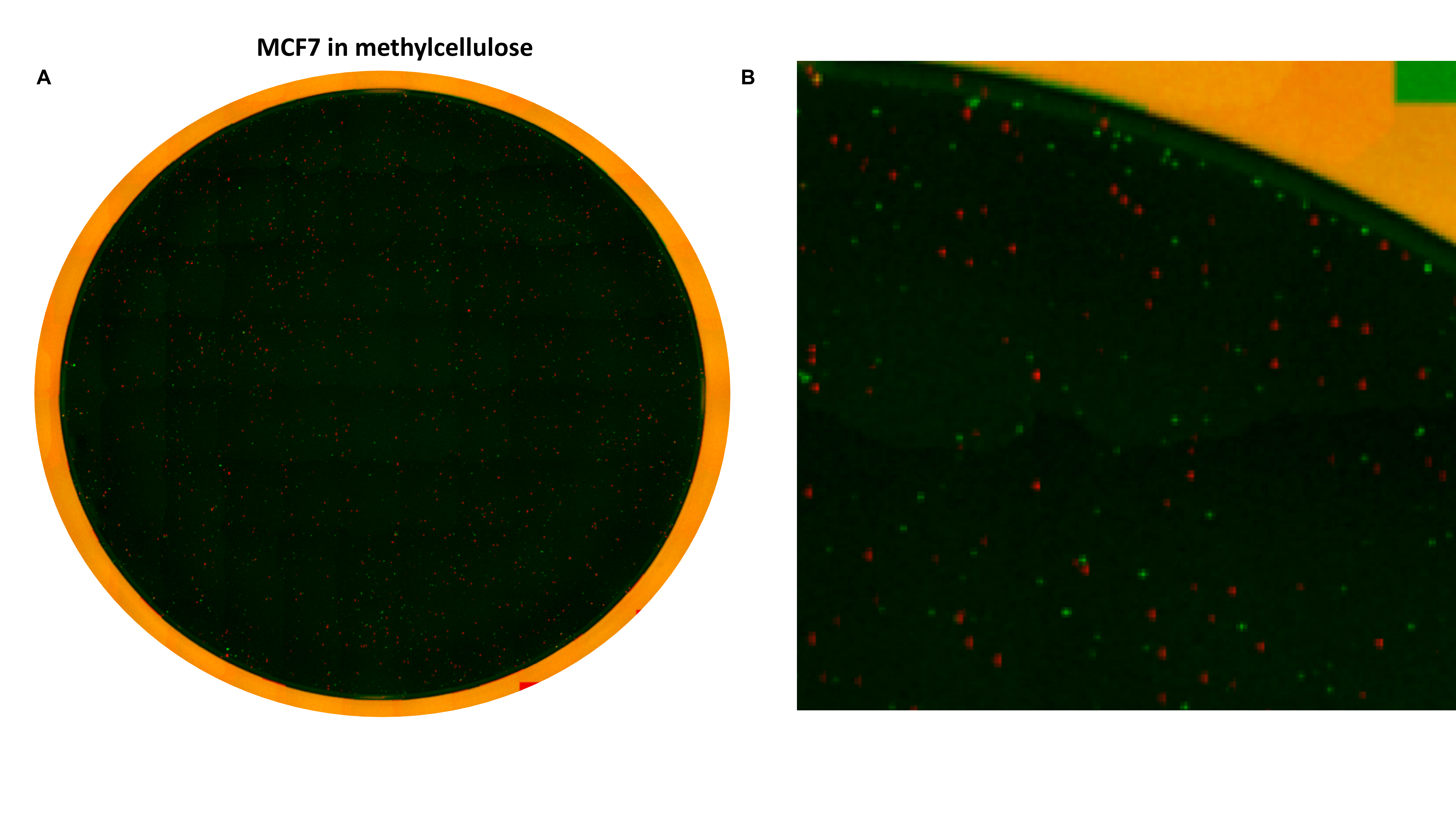


**Supplementary Figure 2:** Cellular drift in methylcellulose. MCF7 cells were seeded at 1000 cells/well in 24 well plates. Mammocult media was supplemented with 5% methylcellulose to reduce aggregation. Images were taken directly after seeding and 24 hours later. (A) Stitched image of entire well. Day 0 (red) and 24 hours (green) are overlaid. (B) Zoomed image of (A) shows that cells at 24 hours (green) have begun to accumulate at the well edge.

**
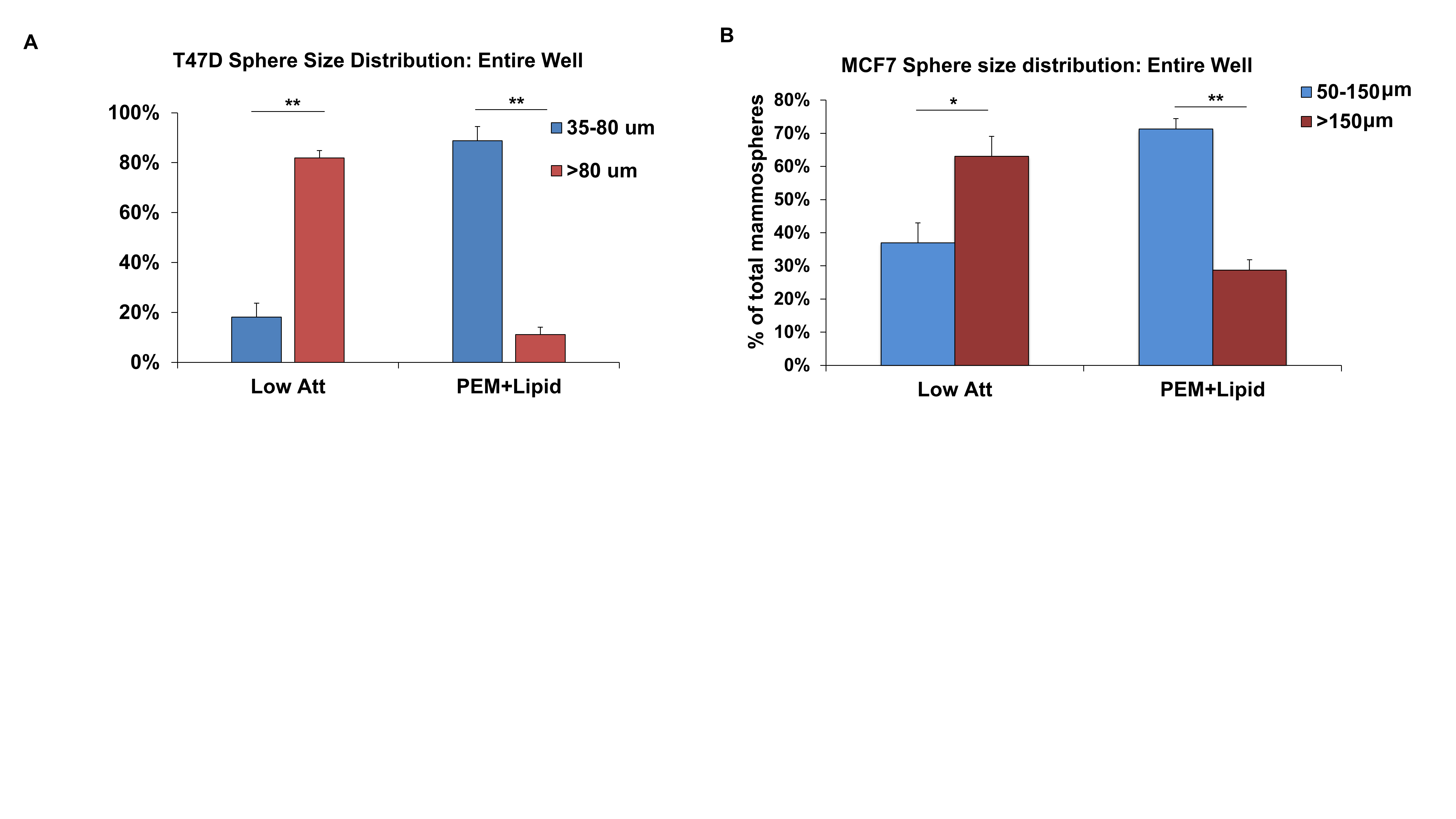
**

**Supplemental Figure 3:** Sphere size analysis for entire well. Related to Figure 3B. A,B) T47D (A) and MCF7 (B) cells were seeded at a density of 1000 cells/well in either low attach or PEM+lipid coated 96 well plates. Sphere sizes were measured and recorded. (Statistics formulated using ANOVA with post-hoc Tukey HSD. *=p value <.01, **=P<.001).

**Supplemental Figure 4:** Secondary sphere formation from lipid tethered primary spheres. PEM+lipid surfaces in 96-well plates were seeded at a density of 1000 cells/well with T47D cells (A) or MCF7 cells (B), and the number of clonal mammospheres counted on Day 7, according to the size thresholds set in Figure 3B. Primary mammospheres were dissociated on Day 7 and used for secondary sphere formation using the same conditions.
